# Japanese subgroup analysis of the phase 3 MONARCH 3 study of abemaciclib as initial therapy for patients with hormone receptor-positive, human epidermal growth factor receptor 2-negative advanced breast cancer

**DOI:** 10.1007/s12282-021-01295-0

**Published:** 2021-10-18

**Authors:** Masato Takahashi, Eriko Tokunaga, Joji Mori, Yoshinori Tanizawa, Jan-Stefan van der Walt, Tsutomu Kawaguchi, Matthew P. Goetz, Masakazu Toi

**Affiliations:** 1 National Hospital Organization Hokkaido Cancer Center, Sapporo, Japan; 2 National Hospital Organization Kyushu Cancer Center, Fukuoka, Japan; 3 Eli Lilly Japan K.K., Kobe, Japan; 4 Eli Lilly and Company, Berkshire, UK; 5 Mayo Clinic, Rochester, MN USA; 6 Breast Cancer Unit, Kyoto University Hospital, Breast Surgery, Graduate School of Medicine, Kyoto University, 54 Shogoin-Kawaracho, Sakyo-ku, Kyoto, 606-8507 Japan

**Keywords:** Abemaciclib, Breast cancer, Cyclin-dependent kinase 4/6, Nonsteroidal aromatase inhibitor

## Abstract

**Background:**

This was a Japanese subpopulation analysis of MONARCH 3, a randomized, double-blind, placebo-controlled phase 3 study of abemaciclib plus nonsteroidal aromatase inhibitors (NSAIs) for initial therapy for advanced breast cancer (ABC).

**Methods:**

Eligibility included postmenopausal women with hormone receptor-positive, human epidermal growth factor receptor 2-negative ABC who had no prior systemic therapy in the advanced disease setting. Patients (*N* = 493) were randomized 2:1 to receive abemaciclib or placebo (150 mg) plus either 1 mg anastrozole or 2.5 mg letrozole (physician’s choice). The primary endpoint was progression-free survival (PFS). Secondary endpoints included objective response rate (ORR), pharmacokinetics (PK), safety, and health-related quality of life (HRQoL).

**Results:**

In Japan, 53 patients were randomized (abemaciclib, *n* = 38; placebo, *n* = 15). At final PFS analysis (November 3, 2017), median PFS was 29.1 and 14.9 months in the abemaciclib and placebo groups, respectively (hazard ratio 0.537; 95% confidence interval 0.224**–**1.289). ORR in measurable disease was 62.1 and 50.0% in the abemaciclib and placebo groups, respectively. The Japanese PK profile was comparable to that of the overall population. Consistent with prior studies, the most frequent adverse events reported were diarrhea (abemaciclib: any grade, 94.7%; grade ≥ 3, 10.5%; placebo: any grade, 46.7%; grade ≥ 3, 0%) and neutropenia (abemaciclib: any grade, 68.4%; grade ≥ 3, 21.1%; placebo: any grade, 0%). HRQoL outcomes were generally similar between treatments except for the diarrhea score, which favored placebo.

**Conclusions:**

Consistent with findings in the overall population, abemaciclib plus NSAI was an effective initial treatment in the Japanese subpopulation, with a manageable safety profile.

**Clinical trial registration:**

NCT02246621; U.S. National Library of Medicine: https://clinicaltrials.gov/ct2/show/NCT02246621.

**Supplementary information:**

The online version contains supplementary material available at 10.1007/s12282-021-01295-0.

## Introduction

Hormone receptor positive (HR+), human epidermal growth factor receptor 2 negative (HER2−) breast cancer comprises over two-thirds of all breast cancers [1]. The mainstay for treatment for this subtype of breast cancer is endocrine therapy (ET), but tumor progression is common due to innate or acquired ET resistance [2]. Recent strategies to prevent or overcome ET resistance have focused on the use of combination cyclin-dependent kinase (CDK) 4/CDK6 inhibitors to target cell cycling pathways. The addition of CDK4/CDK6 inhibitors to ET markedly improves progression-free survival (PFS) over ET alone in patients with HR+, HER2− advanced breast cancer (ABC) and has become the new standard of care in this setting [3].

Abemaciclib is a selective, orally active CDK4/CDK6 inhibitor administered twice daily on a continuous dosing schedule [4–6]. Continuous inhibition of CDK4 and CDK6 by abemaciclib resulted in cellular senescence and apoptosis in human breast cancer cells [6] and had broad antitumor activity in human tumor xenograft models [4, 6]. In phase 3 clinical studies, abemaciclib showed benefit in combination with fulvestrant following progression after initial ET (MONARCH 2) [7, 8] or as first-line therapy in combination with a nonsteroidal aromatase inhibitor (NSAI) (MONARCH 3) [9, 10]. Based on the global MONARCH 2 and MONARCH 3 studies, abemaciclib/ET combination therapy was approved in Japan for the treatment of HR+, HER2− ABC. However, treatment responsiveness is potentially influenced by a variety of interethnic differences in genetics, tumor biology, and drug metabolism [11, 12], and the impact of ethnicity on efficacy and toxicity is not yet well-studied for abemaciclib/ET combination therapies [11, 12].

To gain a better understanding of the response to abemaciclib in Japanese patients with ABC, we evaluated the efficacy and safety outcomes of Japanese patients in MONARCH 2 and MONARCH 3. The MONARCH 2 Japanese subpopulation analysis demonstrated a favorable benefit-risk profile for abemaciclib in combination with fulvestrant in Japanese patients [13]. To explore the response to abemaciclib in Japanese patients more fully, the current study assessed the efficacy and safety of abemaciclib/NSAI combination treatment as initial therapy in the MONARCH 3 Japanese subpopulation. We report here interim and final PFS, safety, health-related quality of life (HRQoL), and pharmacokinetics (PK) outcomes for this subpopulation.

## Patients and methods

### Study design and patients

MONARCH 3 (NCT02246621) was a randomized, double-blind, placebo-controlled, global phase 3 study of abemaciclib plus NSAI (Online Resource 1). Detailed study design and methods for MONARCH 3 have been published [9, 10, 14]. The current analysis was conducted on patients enrolled in MONARCH 3 study sites in Japan. Eligible patients included postmenopausal women ≥ 18 years old, with HR+, HER2− locoregionally recurrent breast cancer (not amenable to curative surgery or radiation therapy) or metastatic breast cancer; an Eastern Cooperative Oncology Group performance status (ECOG PS) ≤ 1; and measurable disease or nonmeasurable bone-only disease per Response Evaluation Criteria In Solid Tumors Version 1.1 (RECIST v. 1.1) [15]. Patients were excluded if they had prior systemic therapy in the advanced disease setting or prior treatment with everolimus or CDK4/CDK6 inhibitors. Prior neoadjuvant/adjuvant ET (e.g., anti-estrogens or aromatase inhibitors) was permitted if patients had a disease-free period > 12 months from treatment completion.

### Treatments and procedures

Patients were randomized 2:1 to receive abemaciclib (150 mg orally, twice daily) plus an NSAI (1 mg anastrozole or 2.5 mg letrozole, orally, once daily) or matching placebo plus an NSAI in 28-day cycles. Randomization to treatment was conducted centrally by the sponsor using a computer-generated random sequence and an interactive web-response system. Patients, study sites, and sponsor study teams were masked to treatment allocation. Randomization was stratified by metastatic site (visceral, bone only, or other) and prior neoadjuvant/adjuvant ET (aromatase inhibitor, no ET, or other). Treatment continued until progressive disease (PD), death, or discontinuation for any other reason. Dose adjustments were permitted for abemaciclib/placebo but not NSAIs (per the label). Crossover of treatment arms was not permitted [9].

### Efficacy and safety assessments

Tumors were imaged by computer tomography or magnetic resonance imaging at baseline, every second cycle for cycles 2 through 18, every third cycle thereafter, and within 2 weeks following clinical progression. Treatment response was assessed by investigators using RECIST v.1.1 [15]. Treatment-emergent adverse events (TEAEs) were summarized using the Medical Dictionary for Regulatory Activities version 20.1 terminology and graded based on the National Cancer Institute Common Terminology Criteria for Adverse Events version 4.0.

### Endpoints

The primary endpoint was investigator-assessed PFS, evaluated from the time of randomization until either death (any cause) or objective PD, as defined by RECIST v.1.1. Secondary efficacy endpoints included objective response rate (ORR; proportion of patients with complete response [CR] or partial response [PR]), disease control rate (DCR; proportion of patients with CR, PR, or stable disease), and clinical benefit rate (CBR; proportion of patients with CR, PR, or stable disease ≥ 6 months).

Additional secondary endpoints included safety, PK, and HRQoL measures. PK samples were planned to be taken from ≥ 150 randomized patients. Samples were collected at prescheduled times on day 1 of cycle 1 (2 to 4 h postdose), cycle 2 (≥ 4.0 h postdose and 7.0 ± 0.5 h postdose), and cycle 3 (predose and 3.0 ± 0.5 h postdose). Concentrations of abemaciclib and its two active metabolites were determined using validated liquid chromatography/tandem mass spectrometry. HRQoL was assessed using the European Organization for Research and Treatment of Cancer Quality of Life Questionnaire-Core 30 (EORTC QLQ-C30) [16] and the EORTC QLQ-Breast Cancer-specific module (EORTC QLQ-BR23) [17], respectively, to assess global health status, functioning, and symptoms. General health status was assessed with the EuroQoL 5-Dimension, 5-level (EQ-5D-5L) instrument, using both the descriptive system (comprising mobility, self-care, usual activities, pain/discomfort, and anxiety/depression dimensions) and the visual analog scale (VAS), which represented self-reported health status on the day of questionnaire completion [18].

### Statistical analyses

Statistical methods for MONARCH 3 have been described [9, 10, 14]. For this subpopulation analysis, *p*-values for comparisons of outcomes between treatments are not reported due to the limited sample size. The primary statistical analysis was the comparison between treatments for PFS and included all patients in the intent-to-treat (ITT) population. Preplanned interim and final PFS analyses were conducted (Online Resource 1). PFS was estimated using the Kaplan–Meier method [19]. The Cox proportional hazard model was used to estimate hazard ratios (HRs) and corresponding 95% confidence intervals (CIs). The data cutoff date for the interim PFS analysis and PK analysis was January 31, 2017. Final PFS, safety, HRQoL, and secondary efficacy endpoints are reported at the data cut-off date of November 3, 2017. ORR, DCR, and CBR were estimated and reported with exact 95% CIs based on normal approximation.

PK analyses were conducted on patients who had plasma PK samples collected and had sufficient dosing information. Mechanistic population PK modeling was used to characterize the PK of abemaciclib in the overall population [20]. Parameter estimates derived from the model, individual dosing histories, and baseline bodyweight were used to simulate individual concentration–time profiles to obtain plasma exposure metrics, including area under the concentration-versus-time curve during one dosing interval at steady state (AUC_τ,ss_), maximum concentration at steady-state (*C*_max,ss_), and minimum/trough concentration at steady state (*C*_min,ss_). Exposure predictions were generated for all patients randomized to receive abemaciclib with and without PK data. Parameter estimates are summarized, comparing the overall and Japanese PK populations.

HRQoL analyses included all patients who completed baseline assessment plus ≥ 1 post-baseline assessment and were conducted as detailed [13, 14] using paper copies of each instrument, which were administered at baseline, every second cycle through cycle 19, and then every third cycle thereafter. Change from baseline scores was assessed using mixed effects-repeated measures models including data and cycles for which ≥ 25% of patients completed questionnaires in both study groups. For EORTC-QLQ-C30 and QLQ-BR23, scoring was from 0 to 100 for each scale; higher scores represented poorer health conditions for symptom scales and better health conditions for global health status and functioning scales. A minimally important difference of ≥ 10-points [21] was used as the threshold for clinically meaningful differences for EORTC outcomes. Each dimension of the EQ-5D-5L descriptive system was scored over 5 severity levels (ranging from “no problem” to “extreme problem”), and an overall index score was derived using the United Kingdom value set (scores ranging from 1 “best possible health” to 0 “death”) [18]. The EQ-5D-5L VAS was scored from 0 (“worst imaginable health status”) to 100 (“best imaginable health status”) [18]. Safety was evaluated in all patients who received ≥ 1 dose of study treatment. Statistical analyses were performed using SAS version 9.2 or later.

## Results

### Patient disposition

In the global MONARCH 3 study, 493 patients were randomly assigned 2:1 to receive abemaciclib plus an NSAI (*n* = 328) or placebo plus an NSAI (*n* = 165) between November 18, 2014, and November 11, 2015 [9]. Of these, 53 patients were enrolled in Japan (abemaciclib, *n* = 38; placebo, *n* = 15; Online Resource 2). At the November 3, 2017, data cut-off, 16 (42.0%) and 5 (33.3%) patients in the abemaciclib and placebo groups of the Japanese subpopulation, respectively, were still on-treatment. The reason for discontinuation of study drug was most frequently due to an adverse event (AE) in the abemaciclib arm (abemaciclib: *n* = 11, 28.9%; placebo: *n* = 0) and PD in the placebo arm (abemaciclib: *n* = 10, 26.3%; placebo: *n* = 10; 66.7%; Online Resource 2).

### Baseline characteristics

Overall, in the Japanese subpopulation, patients had a median age of 64.0 years (minimum–maximum, 47.0**–**75.0 years). Approximately half (45.3%) had visceral disease whereas 26.4% had bone-only disease and 28.3% had other sites of disease. The majority of patients in the Japanese subpopulation had ECOG PS = 0 (83.0%) and progesterone receptor-positive tumors (88.7%). The treatment groups had similar demographic and baseline characteristics in the Japanese subpopulation although a higher proportion in the abemaciclib group had ECOG PS = 1 (abemaciclib 21.1%; placebo 6.7%) and progesterone receptor-positive status (abemaciclib 92.1%; placebo 80.0%; Table 1).

**Table 1:** Baseline demographics and clinical characteristics

Characteristic		Japanese ITT population (*N* = 53)		Overall ITT population (*N* = 493)	
		Abemaciclib + NSAI (*n* = 38)	Placebo + NSAI (*n* = 15)	Abemaciclib + NSAI (*n* = 328)	Placebo + NSAI (*n* = 165)
Age, years	Median (min–max)	63.0 (47**–**75)	64.0 (49**–**72)	63.0 (38**–**87)	63.0 (32**–**88)
Disease setting, *n* (%)	De novo metastatic	0 (0.0)	0 (0.0)	135 (41.2)	61 (37.0)
	Metastatic recurrent	38 (100)	14 (93.3)	182 (55.5)	99 (60.0)
	Locoregional recurrent	0 (0.0)	1 (6.7)	11 (3.4)	5 (3.0)
Prior neoadjuvant/adjuvant therapy, *n* (%)	Aromatase inhibitor	13 (34.2)	5 (33.3)	85 (25.9)	50 (30.3)
	Other ET	9 (23.7)	4 (26.7)	65 (19.8)	30 (18.2)
	None	16 (42.1)	6 (40.0)	178 (54.3)	85 (51.5)
Metastatic site, *n* (%)	Visceral	17 (44.7)	7 (46.7)	172 (52.4)	89 (53.9)
	Bone only	10 (26.3)	4 (26.7)	70 (21.3)	39 (23.6)
	Other	11 (28.9)	4 (26.7)	86 (26.2)	37 (22.4)
Measurable disease, *n* (%)	Yes	29 (76.3)	12 (80.0)	267 (81.4)	130 (78.8)
	No	9 (23.7)	3 (20.0)	61 (18.6)	35 (21.2)
ECOG PS, *n* (%)	0	30 (78.9)	14 (93.3)	192 (58.5)	104 (63.0)
	1	8 (21.1)	1 (6.7)	136 (41.5)	61 (37.0)
PgR, *n* (%)	Positive	35 (92.1)	12 (80.0)	255 (77.7)	127 (77.0)

*ECOG PS* Eastern Cooperative Oncology Group performance status, *ET* endocrine therapy, *ITT* intent-to-treat, *max* maximum, *min* minimum, *N* number of patients in analysis population, *n* number of patients in category or group, *NSAI* nonsteroidal aromatase inhibitor, *PgR* progesterone receptor

The Japanese subpopulation was broadly comparable to the overall MONARCH 3 population for most baseline characteristics (Table 1). However, no Japanese patients (0/53) had de novo metastatic disease compared with 39.8% of the overall population (196/493; Table 1). In addition, a higher proportion of Japanese patients had received prior adjuvant/neoadjuvant ET (31/53; 58.5%) compared with the overall MONARCH 3 population (230/493; 46.7%) whereas a lower proportion (9/53; 17%) had ECOG PS = 1 compared with the overall population (197/493; 40.0%).

### Efficacy

#### PFS

At interim PFS analysis (January 31, 2017; median follow-up time 17.8 months), 18 PFS events (abemaciclib: *n* = 10, 26.3%; placebo: *n* = 8, 53.3%) were observed in the Japanese subpopulation. Median PFS was not reached in the abemaciclib arm and 14.9 months in the placebo arm (HR 0.417; 95% CI 0.152**–**1.146). In comparison, 194 PFS events (abemaciclib: *n* = 108, 32.9%; placebo: *n* = 86, 52.1%) were observed in the overall ITT population, with investigator-assessed median PFS significantly prolonged following abemaciclib (abemaciclib: not reached; placebo: 14.7 months; HR 0.543; 95% CI 0.409**–**0.723; *p* = 0.000021) [9].

**Fig. 1:**
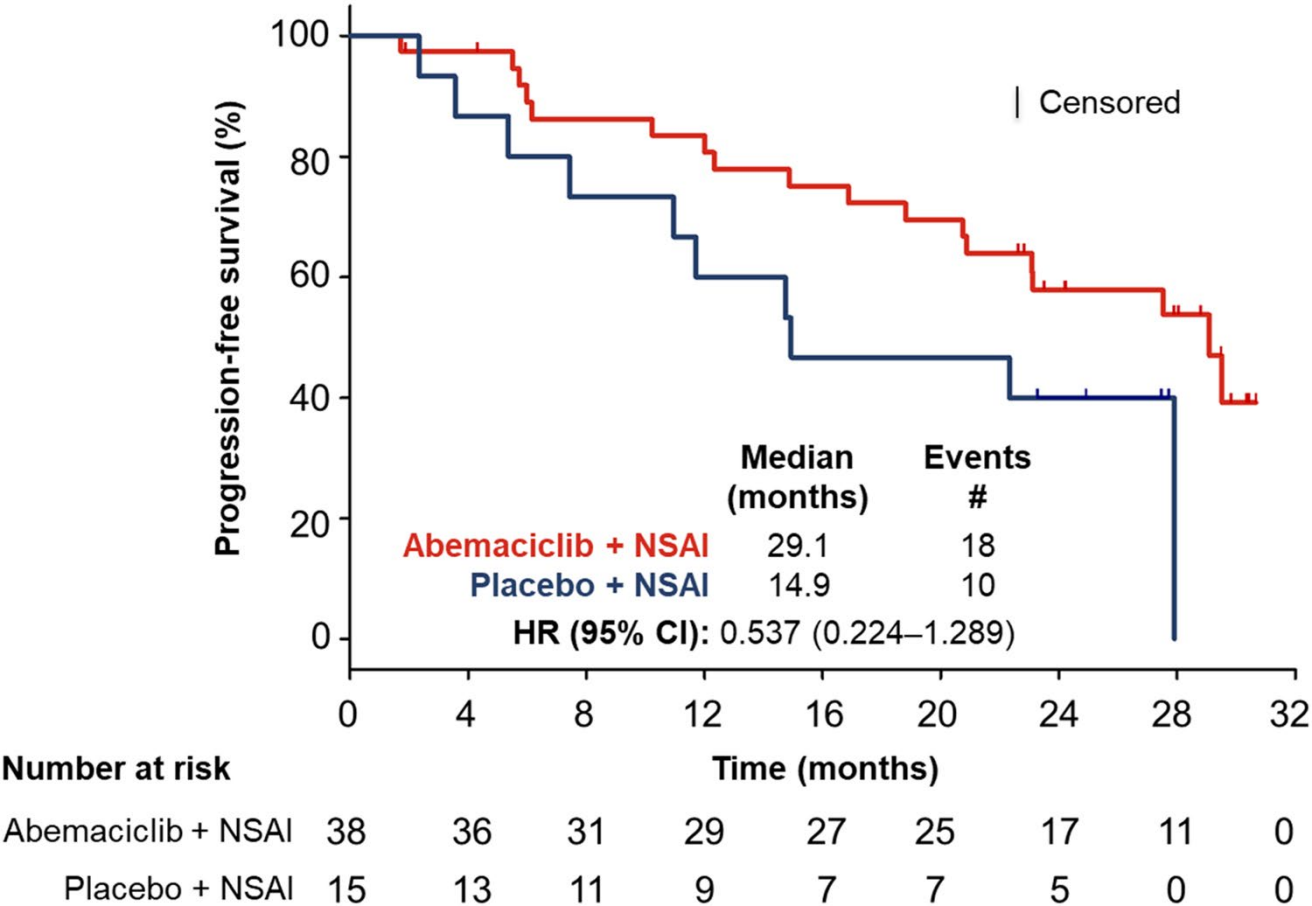
Progression-free survival. PFS analysis at the November 3, 2017 data cut-off date for the MONARCH 3 Japanese subpopulation. PFS was defined as the time from the date of randomization until the date of radiographic documentation of progression, based on investigator assessment, or the date of death, whichever was earlier. The curves and medians (95% CI) were estimated using the Kaplan–Meier method. *CI* confidence interval, *HR* hazard ratio, *n* number of patients in category, *NSAI* nonsteroidal aromatase inhibitor, *PFS* progression-free survival

At the data cut-off date for the final PFS analysis (November 3, 2017; median follow-up time of 26.7 months), 28 PFS events (abemaciclib: *n* = 18, 47.4%; placebo: *n* = 10, 66.7%) were observed in the Japanese subpopulation. The abemaciclib group had a median PFS of 29.1 months compared with 14.9 months in the placebo group (HR 0.537; 95% CI 0.224**–**1.289; Fig. 1). In the overall ITT population, the abemaciclib arm had a final median PFS of 28.2 months compared with 14.8 months median PFS in the placebo arm (HR 0.540; 95% CI 0.418**–**0.698) [10].

#### Objective response rate

In the Japanese subpopulation, the ORR in the abemaciclib arm was 47.4% (95% CI 31.5**–**63.2) across all patients and 62.1% (95% CI 44.4**–**79.7) in patients with measurable disease (Table 2). ORR in the placebo arm was 40.0% (95% CI 15.2**–**64.8) across all patients and 50.0% (95% CI 21.7**–**78.3) in patients with measurable disease. Compared with the placebo group, the abemaciclib group had a lower proportion of patients with PD (Table 2). In comparison, in the overall ITT population, patients with measurable disease had an ORR of 61.0% (95% CI 55.2**–**66.9) in the abemaciclib arm and 45.5% (95% CI 37.0**–**53.9) in the placebo arm [10].

**Table 2:** Summary of best overall response in the Japanese subpopulation of MONARCH 3

Best overall response^a^	All patients				Patients with measurable disease			
	Abemaciclib + NSAI (*n* = 38)		Placebo + NSAI (*n* = 15)		Abemaciclib + NSAI (*n* = 29)		Placebo + NSAI (*n* = 12)	
	*n* (%)	95% CI^b^	*n* (%)	95% CI^b^	*n* (%)	95% CI^b^	*n* (%)	95% CI^b^
Complete response (CR)	0 (0.0)	NA	0 (0.0)	NA	0 (0.0)	NA	0 (0.0)	NA
Partial response (PR)	18 (47.4)	31.5, 63.2	6 (40.0)	15.2, 64.8	18 (62.1)	44.4, 79.7	6 (50.0)	21.7, 78.3
Stable disease (SD)	17 (44.7)	28.9, 60.5	8 (53.3)	28.1, 78.6	8 (27.6)	11.3, 43.9	5 (41.7)	13.8, 69.6
SD persistent for ≥ 6 months	15 (39.5)	23.9, 55.0	7 (46.7)	21.4, 71.9	7 (24.1)	8.6, 39.7	4 (33.3)	6.7, 60.0
Progressive disease (PD)	0 (0.0)	NA	1 (6.7)	− 6.0, 19.3	0 (0.0)	NA	1 (8.3)	− 7.3, 24.0
Objective PD	0 (0.0)	NA	1 (6.7)	− 6.0, 19.3	0 (0.0)	NA	1 (8.3)	− 7.3, 24.0
Not evaluable	3 (7.9)	− 0.7, 16.5	0 (0.0)	NA	3 (10.3)	− 0.7, 21.4	0 (0.0)	NA
Objective response rate (CR + PR)	18 (47.4)	31.5, 63.2	6 (40.0)	15.2, 64.8	18 (62.1)	44.4, 79.7	6 (50.0)	21.7, 78.3
Disease control rate (CR + PR + SD)	35 (92.1)	83.5, 100.7	14 (93.3)	80.7, 106.0	26 (89.7)	78.6, 100.7	11 (91.7)	76.0, 107.3
Clinical benefit rate (CR + PR + SD ≥ 6 months)	33 (86.8)	76.1, 97.6	13 (86.7)	69.5, 103.9	25 (86.2)	73.7, 98.8	10 (83.3)	62.2, 104.4

Data cut-off date: November 3, 2017

*CI*, confidence interval; *n*, number of patients in category or group; *NA*, not applicable; *NSAI*, nonsteroidal aromatase inhibitor; *RECIST*, Response Evaluation Criteria in Solid Tumors

^a^Response was determined by investigators using RECIST version 1.1

^b^CIs were based on normal approximation

### Exposure and pharmacokinetics

Median duration of abemaciclib/placebo was 102.5 and 77.0 weeks in the abemaciclib and placebo groups, respectively (anastrozole 125.1 and 64.9 weeks, respectively; letrozole 58.4 and 77.0 weeks, respectively; Online Resource 3). The median dose intensity for abemaciclib was 206.4 mg/day, and median relative dose intensity was 68.8%.

**Fig. 2:**
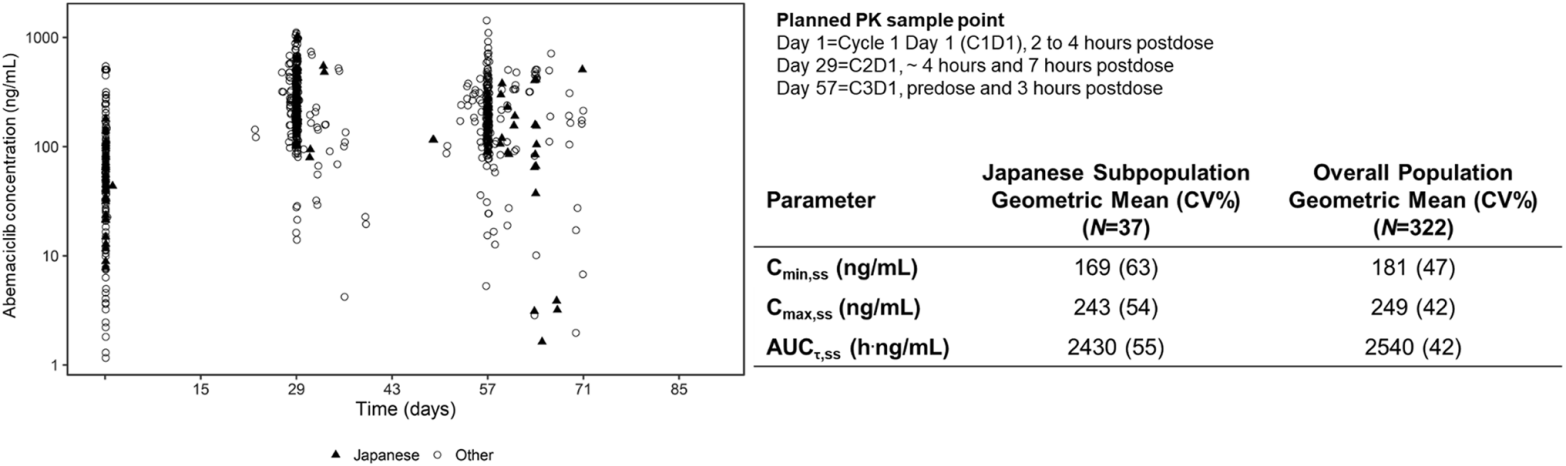
Pharmacokinetic analysis in patients receiving abemaciclib plus an NSAI. Blood samples were obtained at prescheduled times on day 1 of cycles 1**–**3. Plasma concentrations of abemaciclib for individual patients over the course of the analysis are shown in the top graph (triangles indicate Japanese patients, *N* = 38; circles indicate other patients in the global population, *N* = 166). Geometric mean trough and peak concentrations (CV%) for the Japanese subpopulation and MONARCH 3 study population are summarized in the table. Exposure predictions were generated for patients randomized to the abemaciclib arm with and without PK data (*N* = 322) (due to technical issues, PK parameters could not be obtained to simulate exposure for 1 patient in the Japanese subpopulation and 3 patients in the global population). *AUC*_*τ,ss*_ area under the concentration versus time curve during one dosing interval at steady state, *C*_*max,ss*_ maximum concentration at steady-state, *C*_*min,ss*_ minimum/trough concentration at steady state, *CV* coefficient of variation, *NSAI* nonsteroidal aromatase inhibitor, *PK* pharmacokinetics

Abemaciclib plasma concentrations for individual patients over the study period, PK steady state exposure metrics (AUC_τ,ss_, *C*_max,ss_, *C*_min,ss_), and inter-individual variability were similar for patients in the Japanese PK subpopulation and overall MONARCH 3 PK population (Fig. 2).

### Safety

At final PFS analysis (November 3, 2017), all patients in the Japanese subpopulation reported ≥ 1 TEAE, with a higher proportion of patients in the abemaciclib group reporting ≥ grade 3 TEAEs (grade 3, *n* = 22, 57.9%; grade 4, *n* = 4, 10.5%) compared with the placebo group (grade 3, *n* = 3, 20.0%; grade 4, *n* = 1, 6.7%). The rates of any grade, grade 3, and grade 4 TEAEs in the Japanese subpopulation were consistent with those of the overall safety population (abemaciclib: any grade, *n* = 323, 98.8%; grade 3, *n* = 169, 51.7%; grade 4, *n* = 22; 6.7%; placebo: any grade, *n* = 152, 94.4% grade 3, *n* = 36, 22.4%; grade 4, *n* = 4, 2.5%) [10]. No grade 5 TEAEs were reported in the Japanese subpopulation, whereas the overall safety population reported grade 5 TEAEs in both treatment arms (abemaciclib: *n* = 11; 3.4%; placebo: *n* = 2; 1.2%) [10].

The most common TEAEs in the abemaciclib group in the Japanese subpopulation were diarrhea (abemaciclib: any grade, 94.7%; grade ≥ 3, 10.5%; placebo: any grade, 46.7%; grade ≥ 3, 0%; Table 3) and neutropenia (abemaciclib: any grade, 68.4%; grade ≥ 3, 21.1%; placebo: any grade, 0%; grade ≥ 3, 0%), which were reported at a higher frequency in the abemaciclib group than in the placebo group. Grades 1–2, but not grade ≥ 3, diarrhea and neutropenia were reported more frequently in both treatment groups of the Japanese subpopulation compared to the overall safety population (diarrhea: abemaciclib: any grade, 82.3%; grade ≥ 3, 9.5%; placebo: any grade, 32.3%; grade ≥ 3, 1.2%; neutropenia: abemaciclib: any grade, 43.7%; grade ≥ 3, 23.9%; placebo: any grade, 1.9%; grade ≥ 3, 1.2%) [10]. Incidence of any grade and ≥ grade 3 TEAEs of increased alanine aminotransferase (ALT)/aspartate aminotransferase (AST) were also higher in both treatment arms of the Japanese subpopulation (ALT increased: abemaciclib: any grade, 50.0%; grade ≥ 3, 26.3%; placebo: any grade, 20.0%; grade ≥ 3, 0%); AST increased: abemaciclib: any grade, 50.0%; grade ≥ 3, 15.8%; placebo: any grade, 13.3%; grade ≥ 3, 0%) compared with the overall safety population (ALT increased: abemaciclib: any grade, 17.4%; grade ≥ 3, 6.4%; placebo: any grade, 7.5%; grade ≥ 3, 1.9%; AST increased: abemaciclib: any grade, 16.8%; grade ≥ 3, 3.7%; placebo: any grade, 7.5%; grade ≥ 3, 1.2%) [10]). In contrast, fatigue (any grade) was less frequent in the Japanese subpopulation (abemaciclib: any grade, 13.2%; grade ≥ 3, 0%; placebo: any grade, 13.3%; grade ≥ 3, 0%) compared to the overall safety population (abemaciclib, any grade, 41.3%; grade ≥ 3, 1.8%; placebo, any grade, 33.5%; grade ≥ 3, 0%; [10]). Pneumonitis (interstitial lung disease [ILD]), a potentially serious TEAE, was reported at a higher rate in the abemaciclib arm of the Japanese subpopulation compared with placebo (abemaciclib: any grade, *n* = 4, 10.5%, grade ≥ 3, *n* = 1, 2.6%; placebo: any grade, *n* = 1, 6.7%; grade ≥ 3, 0%; overall safety population: abemaciclib: any grade, *n* = 11, 3.4%; grade ≥ 3, *n* = 2; 0.6%; placebo: any grade, *n* = 2, 1.2%; grade ≥ 3, 0%).

**Table 3:** Treatment-emergent adverse events occurring in ≥ 20% of Japanese patients in the abemaciclib arm by grade

≥ 20% in abemaciclib arm, *n* (%)	Abemaciclib + NSAI (*n* = 38)				Placebo + NSAI (*n* = 15)			
	Grade 1	Grade 2	Grade 3	Grade 4	Grade 1	Grade 2	Grade 3	Grade 4
Diarrhea	23 (60.5)	9 (23.7)	4 (10.5)	0 (0.0)	6 (40.0)	1 (6.7)	0 (0.0)	0 (0.0)
Neutropenia	4 (10.5)	14 (36.8)	8 (21.1)	0 (0.0)	0 (0.0)	0 (0.0)	0 (0.0)	0 (0.0)
Leukopenia	4 (10.5)	9 (23.7)	8 (21.1)	0 (0.0)	1 (6.7)	1 (6.7)	0 (0.0)	1 (6.7)
ALT increased	7 (18.4)	2 (5.3)	9 (23.7)	1 (2.6)	2 (13.3)	1 (6.7)	0 (0.0)	0 (0.0)
Anemia	6 (15.8)	9 (23.7)	4 (10.5)	0 (0.0)	0 (0.0)	1 (6.7)	0 (0.0)	0 (0.0)
AST increased	10 (26.8)	3 (7.9)	6 (15.8)	0 (0.0)	2 (13.3)	0 (0.0)	0 (0.0)	0 (0.0)
Alopecia	13 (34.2)	2 (5.3)	0 (0.0)	0 (0.0)	3 (20.0)	0 (0.0)	0 (0.0)	0 (0.0)
Nausea	12 (31.6)	2 (5.3)	0 (0.0)	0 (0.0)	4 (26.7)	0 (0.0)	0 (0.0)	0 (0.0)
Dysgeusia	8 (21.1)	3 (7.9)	0 (0.0)	0 (0.0)	0 (0.0)	0 (0.0)	0 (0.0)	0 (0.0)
Vomiting	11 (28.9)	0 (0.0)	0 (0.0)	0 (0.0)	6 (40.0)	0 (0.0)	0 (0.0)	0 (0.0)
Abdominal pain	8 (21.1)	1 (2.6)	0 (0.0)	0 (0.0)	0 (0.0)	0 (0.0)	0 (0.0)	0 (0.0)
Blood creatinine increased	5 (13.2)	4 (10.5)	0 (0.0)	0 (0.0)	0 (0.0)	0 (0.0)	0 (0.0)	0 (0.0)
Decreased appetite	6 (15.8)	2 (5.3)	1 (2.6)	0 (0.0)	0 (0.0)	0 (0.0)	1 (6.7)	0 (0.0)
Fatigue	4 (10.5)	1 (2.6)	0 (0.0)	0 (0.0)	0 (0.0)	2 (13.3)	0 (0.0)	0 (0.0)
Rash	7 (18.4)	2 (5.3)	0 (0.0)	0 (0.0)	0 (0.0)	0 (0.0)	1 (6.7)	0 (0.0)
Headache	7 (18.4)	1 (2.6)	0 (0.0)	0 (0.0)	2 (13.3)	0 (0.0)	0 (0.0)	0 (0.0)
Pruritis	8 (21.1)	0 (0.0)	0 (0.0)	0 (0.0)	1 (6.7)	0 (0.0)	0 (0.0)	0 (0.0)

Data cutoff date: November 3, 2017

MedDRA version 20.1; CTCAE version 4

*ALT* alanine aminotransferase, *AST* aspartate aminotransferase, *CTCAE* Common Terminology Criteria for Adverse Events, *MedDRA* Medical Dictionary for Regulatory Activities, *n* number of patients, *NSAI* nonsteroidal aromatase inhibitor

Online Resource 4 summarizes dose adjustments in MONARCH 3. In the Japanese subpopulation, the rates of dose reductions and omissions of abemaciclib due to AEs were 55.3% and 73.7%, respectively (placebo, 6.7% and 33.3%, respectively). AEs leading to abemaciclib dose adjustments in the Japanese subpopulation were most commonly increased ALT (dose reduction: 15.8%; dose omission: 21.1%), neutropenia (dose reduction: 13.2%; dose omission: 18.4%), and diarrhea (dose reduction: 13.2%; dose omission: 13.2%). In the overall safety population, dose adjustments due to neutropenia (dose reduction: 12.8%; dose omission: 17.4%) and diarrhea (dose reduction: 13.8%; dose omission: 15.3%) occurred at similar rates to those of the Japanese subpopulation, but dose adjustments due to hepatic events were infrequent (Online Resource 4).

The rates of discontinuation of any study drug due to an AE in the abemaciclib group were 34.2% and 25.1% in the Japanese subpopulation and overall safety population, respectively. Within the abemaciclib group of the Japanese subpopulation, discontinuation of any study drug was most commonly due to AEs of increased ALT (*n* = 4; 10.5%) or AST (*n* = 2; 5.3%), with 1 patient (2.6%) each discontinuing due to AEs of neutropenia and diarrhea. The rates of discontinuation of any study drug due to diarrhea and neutropenia in the overall population were similar to those in the Japanese subpopulation, whereas the overall population had fewer discontinuations of any study drug due to increased ALT (*n* = 7; 2.1%) or AST (*n* = 2; 0.6%) (Online Resource 4).

### Health-related quality of life

Baseline EORTC QLQ-C30 global health status score and EORTC QLQ-C30 and QLQ-BR23 functional and symptom scores were similar across treatment groups except for financial difficulties, which were reported more frequently in the abemaciclib arm (Table 4). Differences between treatment arms in the change from baseline over the treatment course for assessment items on the EORTC QLQ-C30 and QLQ-BR23 generally did not meet the threshold for a clinically meaningful difference. The exception was the QLQ-C30 diarrhea score (mean [standard error]: abemaciclib, 24.9 [2.5]; placebo, 2.8 [4.0]), which favored the placebo arm. EQ-5D-5L index and VAS scores were also similar across treatment groups (Online Resource 5).

**Table 4:** Mean baseline scores and within-treatment group change from baseline: EORTC QLQ-C30 and QLQ-BR23

Assessment	Baseline score Mean (SD)		Change from baseline^a^ Least squares mean (SE)	
	Abemaciclib + NSAI (*n* = 36)	Placebo + NSAI (*n* = 15)	Abemaciclib + NSAI (*n* = 36)	Placebo + NSAI (*n* = 15)
EORTC QLQ-C30^b^				
Global health status	65.7 (25.3)	70.6 (20.6)	− 7.1 (2.4)	− 3.5 (3.7)
Functional scales				
Physical	81.4 (19.3)	80.0 (15.3)	− 1.2 (1.8)	2.7 (2.8)
Role	83.3 (25.8)	83.3 (19.9)	− 6.4 (2.8)	1.4 (4.4)
Emotional	78.0 (19.6)	82.8 (13.5)	3.7 (2.3)	− 0.1 (3.5)
Cognitive	82.4 (18.2)	87.8 (18.3)	− 3.9 (2.5)	− 8.4 (3.8)
Social	84.3 (24.2)	88.9 (15.0)	0.5 (2.2)	3.5 (3.4)
Symptom scales				
Fatigue	27.8 (23.4)	24.4 (14.7)	8.5 (2.6)	3.6 (3.9)
Nausea and vomiting	2.8 (10.2)	1.1 (4.3)	2.8 (1.7)	4.8 (2.6)
Pain	26.4 (26.5)	20.0 (14.4)	− 4.0 (2.7)	− 2.6 (4.1)
Dyspnea	13.9 (21.6)	11.1 (20.6)	10.7 (3.4)	3.1 (5.1)
Insomnia	24.1 (23.4)	17.8 (24.8)	− 8.1 (2.8)	− 6.3 (4.4)
Appetite loss	10.2 (17.5)	15.6 (21.3)	3.3 (2.5)	− 0.3 (3.8)
Constipation	15.7 (23.2)	6.7 (18.7)	− 1.2 (2.2)	1.0 (3.4)
Diarrhea	3.7(10.6)	6.7 (13.8)	24.9 (2.5)	2.8 (4.0)
Financial difficulties	18.5 (29.2)	6.7 (13.8)	− 2.0 (2.1)	− 4.5 (3.3)
EORTC QLQ-BR23^b^ functional scales				
Body image	78.7 (23.3)	84.5 (14.0)	− 4.5 (2.9)	− 7.7 (4.4)
Sexual functioning	5.1 (12.5)	0.0 (0.0)	− 1.0 (0.7)	− 1.6 (1.1)
Future perspectives	50.9 (37.8)	55.6 (30.0)	3.2 (3.7)	− 0.9 (5.7)
Symptom scales				
Systemic therapy side effects	12.0 (9.1)	12.1 (10.0)	7.8 (1.9)	7.3 (2.9)
Breast	20.8 (18.0)	14.5 (15.3)	− 9.4 (1.6)	− 7.2 (2.4)
Arm	17.3 (24.4)	23.0 (22.4)	1.1 (2.3)	1.8 (3.5)

Data cut-off date: November 3, 2017

*EORTC* European Organization for Research and Treatment of Cancer; *MMRM* mixed model-repeated measures; *n* number of subjects in the population with baseline and post-baseline value for the question at the specified visit; *NSAI* nonsteroidal aromatase inhibitor; *QLQ-BR23* Quality of Life Questionnaire-Breast subscale, 23 items; *QLQ-C30* Quality of Life Questionnaire-Core 30; *SD* standard deviation; *SE* standard error

^a^Change from baseline was assessed across all postbaseline visits with a Type 3 sums of squares MMRM model (Change from Baseline = Treatment + Visit + Treatment*Visit + Baseline), including all cycles for which at least 25% of patients in each group have an assessment for each of the functional and symptom scales. Unstructured covariance structure was used for the MMRM model

^b^Deterioration of symptoms is represented by an increase in scores; deterioration of global health status and functioning scores is represented by a decrease in scores

## Discussion

The results of this subpopulation analysis of MONARCH 3 indicate that abemaciclib/NSAI combination therapy is an effective initial treatment with a manageable safety profile in postmenopausal Japanese women with HR+, HER2− ABC. At the interim analysis, the median PFS for the Japanese subpopulation (abemaciclib: not reached; placebo: 14.9 months; HR 0.417; 95% CI 0.152**–**1.146) was consistent with that of the overall ITT analysis, which showed significant benefit from the addition of abemaciclib to NSAI treatment (abemaciclib: not reached; placebo: 14.7 months; HR 0.543; 95% CI 0.409**–**0.723; *p* = 0.000021) [9]. At final analysis, abemaciclib plus NSAI treatment resulted in an improvement in median PFS by 14.2 months over NSAI alone in the Japanese subpopulation (abemaciclib 29.1 months; placebo 14.9 months; HR 0.537; 95% CI 0.224**–**1.289) and 13.4 months in the overall MONARCH 3 population (abemaciclib 28.2 months; placebo 14.8 months; HR 0.540; 95% CI 0.418**–**0.698) [10]. In patients with measurable disease, abemaciclib/NSAI combination therapy showed greater antitumor activity compared to NSAI alone, both in the Japanese subpopulation (ORR: abemaciclib, 62.1% [95% CI 44.4**–**79.7]; placebo 50.0% [95% CI 21.7**–**78.3]) and in the overall ITT population (ORR: abemaciclib, 61.0% [95% CI 55.2**–**66.9]; placebo, 45.5% [95% CI 37.0**–**53.9]) [10]. Median overall survival was not reached in either treatment group of the Japanese subpopulation and overall ITT population, and the data were judged immature (data not shown), indicating that further follow-up is required.

Mean abemaciclib exposure was similar in the Japanese subpopulation and overall MONARCH 3 population, with overlapping plasma concentrations ranges, indicating that the pharmacokinetic profiles of abemaciclib were consistent between the two PK populations. Abemaciclib/NSAI combination treatment had a generally tolerable safety profile in Japanese patients with ABC. Although grade ≥ 3 TEAEs occurred in 68.4% of Japanese patients in the abemaciclib arm, these were predominantly grade 3 in severity and manageable with dose adjustments and supportive care, with no grade 5 events. The Japanese subpopulation safety profile was broadly consistent with that of the overall MONARCH 3 population, with a similar incidence of any grade, grade 3, and grade 4 TEAEs. Diarrhea and neutropenia were the most common TEAEs in the abemaciclib arm of both the Japanese subpopulation and overall safety population. Although the incidence of grade 1**–**2 diarrhea and neutropenia was higher in the Japanese subpopulation compared to the overall safety population, the rates of dose adjustments and discontinuations due to these TEAEs were similar between the two populations. Compared to the overall safety population, the abemaciclib arm of the Japanese subpopulation also had higher rates of increased ALT/AST, including grade ≥ 3 events, resulting in a higher rate of discontinuation of any study treatment due to these TEAEs.

ILD is a potentially serious complication of many therapeutic agents and is a class side effect of CDK4/CDK6 inhibitors [22]. In the current study, the incidence of ILD was higher following abemaciclib treatment in the Japanese subpopulation (any grade, *n* = 4, 10.5%; grade ≥ 3, *n* = 1; 2.6%) compared to the overall safety population (any grade, *n* = 11; 3.4%; grade ≥ 3, *n* = 2; 0.6%). This finding, and the recent report on the incidence for ILD in abemaciclib-treated Japanese patients in the real-world setting [23], highlight the need for healthcare providers to recognize the potential for ILD in abemaciclib-treated patients and to monitor regularly for symptoms of ILD during abemaciclib treatment.

Change from baseline in HRQoL assessment items was generally similar between treatment groups in the Japanese subpopulation. The exception to this was the EORTC QLQ-C30 diarrhea score, which was numerically lower in the placebo group, meeting the threshold for a clinically meaningful difference. This finding is consistent with the higher incidence of TEAEs of diarrhea reported in abemaciclib-treated patients compared with the placebo group. Diarrhea was generally manageable by dose reduction/omission and supportive treatment in the MONARCH 3 Japanese subpopulation with only one patient discontinuing due to an AE of diarrhea. The current results indicate that the addition of abemaciclib to an NSAI did not result in a clinically meaningful decline in global health status, functional scales, and most of the symptom scales in women with HR+, HER2− ABC compared to NSAI alone. These findings are consistent with the HRQoL and safety findings in the overall MONARCH 3 study population and more broadly with those of the abemaciclib clinical program [7–10, 13, 14, 24–26].

Overall, the current findings agree with and build upon those from the MONARCH 2 Japanese subpopulation analyses. MONARCH 2 examined abemaciclib in combination with fulvestrant in patients with HR+, HER2− ABC who had progressed on prior ET and who were of any menopausal status, representing a younger patient population (median age [minimum–maximum]: 58.0 [32–81] years) [13] compared with the MONARCH 3 subpopulation (64.0 [47–75] years). In Japanese patients from both studies, abemaciclib conferred a PFS benefit (MONARCH 2: 6.9 months; MONARCH 3: 14.2 months) and improved the ORR over the placebo controls (MONARCH 2: abemaciclib, 37.5%; placebo, 12.9%; MONARCH 3: abemaciclib, 62.1%; placebo, 50.0%) [13]. Japanese PK, safety, and HRQoL profiles were consistent with each other and in accordance with those of the respective overall study populations [7–10, 13, 14]. Furthermore, both MONARCH 2 and MONARCH 3 Japanese subpopulations experienced a higher rate of any grade and grade ≥ 3 hematologic events and increased ALT/AST than the overall safety population [7, 8, 13], identifying clinically important toxicities for abemaciclib-treated Japanese patients.

A major limitation of this analysis is the small sample size of the Japanese subpopulation, and statistical hypothesis testing was not performed. In addition, differences in baseline demographic/clinical characteristics have the potential to affect response to treatment. These included differences between treatment groups in the Japanese subpopulation (e.g., a higher proportion of patients had ECOG PS = 1 and progesterone receptor-positive status in the abemaciclib arm) and between the Japanese subpopulation and the overall population (e.g., no Japanese patients had de novo metastatic disease compared with nearly 40% in the overall population; 58.5% in the Japanese subpopulation had received prior ET compared with 46.7% in the overall population).

## Conclusion

The results of this Japanese subpopulation analysis indicate that, compared to NSAI alone, abemaciclib-NSAI combination therapy improved clinical outcomes with an acceptable safety profile when used as an initial treatment in postmenopausal women with advanced, ET-resistant HR+, HER2− breast cancer. The MONARCH 2 and MONARCH 3 Japanese subpopulation analyses add to the limited information available on abemaciclib in Japanese patients, indicating that abemaciclib/ET therapies are efficacious in either the first- or second-line setting in HR+, HER2− ABC. For both studies, efficacy and safety findings of the Japanese subpopulations were broadly consistent with those of the overall populations, but the higher incidence of some TEAEs (e.g., increased ALT/AST) in both Japanese subpopulations highlight potential country-specific clinical management considerations.

## Supplementary information

Below is the link to the electronic supplementary material.

Supplementary file1 (PDF 580 kb)
