# Supplementary material for: Japanese subgroup analysis of the phase 3 MONARCH 3 study of abemaciclib as initial therapy for patients with hormone receptor-positive, human epidermal growth factor receptor 2-negative advanced breast cancer
Source: Breast Cancer. 2021 Oct 18;29(1):174–84. doi: 10.1007/s12282-021-01295-0 (PMC8732856; doi:10.1007/s12282-021-01295-0)
Supplement: Supplementary file 1 — Supplementary file1 (PDF 580 kb) [file 12282_2021_1295_MOESM1_ESM.pdf]

Online resources for:

Breast Cancer

Japanese subgroup analysis of the phase 3 MONARCH 3 study of abemaciclib as initial therapy for patients with hormone receptor-positive, human epidermal growth factor receptor 2-negative breast cancer

Masato Takahashi, Eriko Tokunaga, Joji Mori, Yoshinori Tanizawa, Jan-Stefan van der Walt, Tsutomu Kawaguchi, Matthew P. Goetz, Masakazu Toi

Corresponding author:

Masakazu Toi

Kyoto University Hospital, Kyoto, Japan

Email: toi@kuhp.kyoto-u.ac.jp

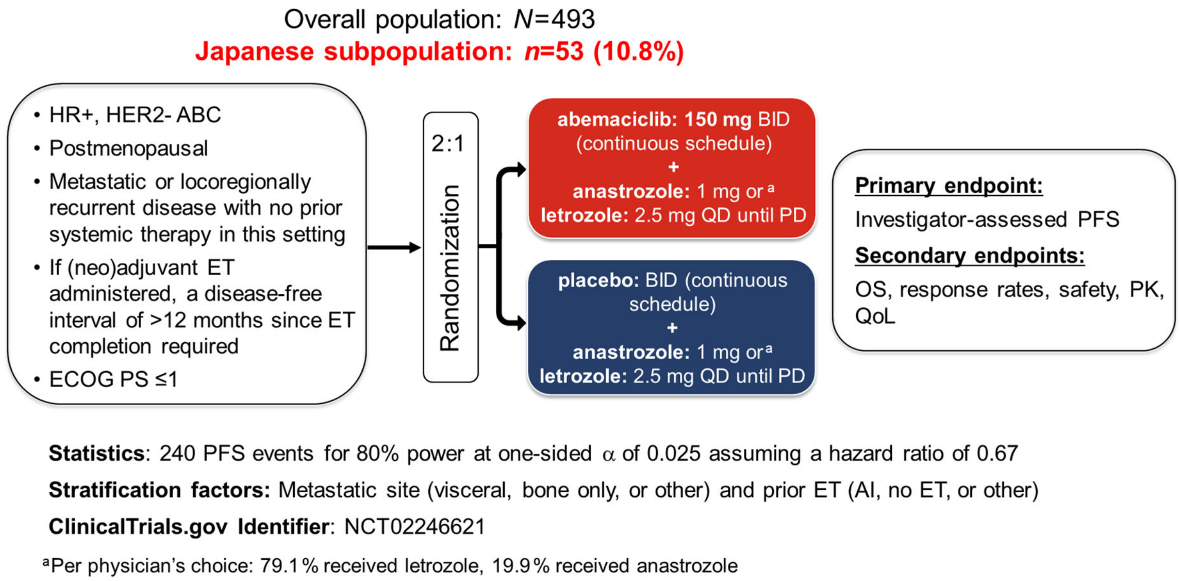

Online Resource 1: MONARCH 3 study design

MONARCH 3 was a randomized, double-blind, placebo-controlled, global phase 3 study of abemaciclib plus an NSAI in women with HR+, HER2- locally advanced or metastatic breast cancer. Patients were randomized 2:1 to receive abemaciclib (150 mg orally, twice daily) or matching placebo plus an NSAI (either 1 mg anastrozole or 2.5 mg letrozole, orally, once daily) in 28-day cycles, stratified by metastatic site (visceral, bone only, or other) and prior neoadjuvant/adjuvant ET (AI, no ET, or other). The primary endpoint was investigator-assessed PFS, and key secondary endpoints included overall survival, best overall response, safety, PK, and HRQoL. Final PFS analysis was preplanned to occur at approximately 240 PFS events in the overall ITT population, for 80% power with a one-sided alpha = 0.025 assuming a hazard ratio of 0.67 in favor of the abemaciclib arm. Interim PFS analysis was preplanned to occur at approximately 189 PFS events in the overall ITT population, assuming a hazard ratio of <0.56 (two-sided, $p<0.0005$).

ABC, advanced breast cancer; *AI*, aromatase inhibitor; *BID*, twice daily dose; *ECOG PS*, Eastern Cooperative Oncology Group performance status; *ET*, endocrine therapy; *HER2-*, human epidermal growth factor receptor 2 negative; *HR*, hazard ratio; *HR+*, hormone receptor-positive; *ITT*, intent to treat; *N*, number of patients in analysis population; n, number of patients in category or group; *NSAI*, nonsteroidal aromatase inhibitor; *OS*, overall survival; *PD*, progressive disease; *PFS*, progression-free survival; *PK*, pharmacokinetics; *QD*, every day; *HRQoL*, quality of life

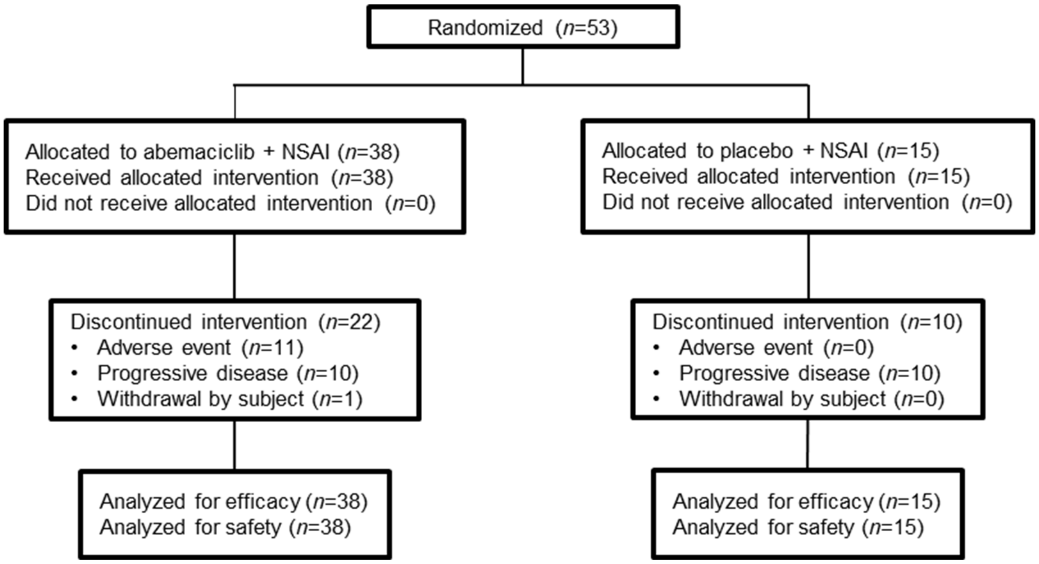

Online Resource 2: Patient disposition Data cut-off date: November 3, 2017

n, number of patients

Online Resource 3. Exposure summary in the MONARCH 3 Japan subpopulation

	**Abemaciclib + NSAI (*n*=38)**	**Placebo + NSAI (*n*=15)**
**Duration of therapy (abemaciclib), weeks**		
Mean (SD)	79.3 (52.8)	76.2 (43.5)
Median	102.5	77.0
IQR	20.10 – 129.1	32.9 – 121.0
**Duration of therapy (anastrozole), weeks**		
Mean (SD)	123.4 (8.0)	66.7 (50.3)
Median	125.1	64.9
IQR	118.0 – 140130.0	24.4 – 109.0
**Duration of therapy (letrozole), weeks**		
Mean (SD)	66.8 (53.1)	79.7 (42.9)
Median	58.4	77.0
IQR	5.9 – 140.0	50.1 – 127124.1
**Dose intensity[a] (abemaciclib), mg per day**		
Mean (SD)	210.3 (59.8)	290.2 (15.8)
Median	206.4	295.6
IQR	177.9 – 248.4	291.2 – 298.7
**Relative dose intensity[b] (abemaciclib), %**		
Mean (SD)	70.1 (19.9)	96.7 (5.3)
Median	68.8	98.5
IQR	59.3 – 82.8	97.1 – 99.6

Data cut-off date November 3, 2017

[a]Dose intensity refers to the actual total amount of drug administered per day

[b]Relative dose intensity refers to the percentage of actual amount of drug taken relative to amount of drug prescribed

n, number of patients in category or group; *IQR*, interquartile range; *NSAI*, nonsteroidal aromatase inhibitor; *SD*, standard deviation

Online Resource 4. Dose adjustment summary for the MONARCH 3 Japan subpopulation and overall population

	**Japanese safety population**		**Overall safety population**	
	**Abemaciclib + NSAI (*n*=38)**	**Placebo + NSAI (*n*=15)**	**Abemaciclib + NSAI (*N*=327)**	**Placebo + NSAI (*N*=161)**
**Patients with ≥1 dose adjustment (abemaciclib/placebo)**	31 (81.6)	5 (33.3)	220 (67.3)	47 (29.2)
**Dose reductions due to AE, *n* (%)**	21 (55.3)	1 (6.7)	152 (46.5)	10 (6.2)
Reasons, ≥10% in abemaciclib arm of Japanese subpopulation				
ALT increased	6 (15.8)	0 (0.0)	8 (2.4)	1 (0.6)
Neutropenia	5 (13.2)	0 (0.0)	42 (12.8)	1 (0.6)
Diarrhea	5 (13.2)	0 (0.0)	45 (13.8)	3 (1.9)
**Dose omissions due to AE, *n* (%)**	28 (73.7)	5 (33.3)	197 (60.2)	33 (20.5)
Reasons, ≥10% in abemaciclib arm of Japanese subpopulation				
ALT increased	8 (21.1)	0 (0.0)	17 (5.2)	3 (1.9)
Neutropenia	7 (18.4)	0 (0.0)	57 (17.4))	1 (0.6)
Diarrhea	5 (13.2)	0 (0.0)	50 (15.3)	3 (1.9)
**Discontinuation of any study drug due to AE, *n* (%)**	13 (34.2)	0 (0.0)	82 (25.1)	7 (4.3)
Reasons[a]				
ALT increased	4 (10.5)	0 (0.0)	7 (2.1)	0 (0.0)
AST increased	2 (5.3)	0 (0.0)	2 (0.6)	0 (0.0)
Neutropenia	1 (2.6)	0 (0.0)	9 (2.8)	0 (0.0)
Diarrhea	1 (2.6)	0 (0.0)	6 (1.8)	0 (0.0)

Data cut-off date November 3, 2017.

[a]Only data for ALT/AST increased, diarrhea, and neutropenia are presented.

AE, adverse events; *ALT*, alanine aminotransferase; *AST*, aspartate aminotransferase; *N*, number of patients in analysis population; n, number of patients in category or group; *NSAI*, nonsteroidal aromatase inhibitor.

Online Resource 5. Mean EQ-5D-5L descriptive system and VAS scores in MONARCH 3 Japanese subpopulation

	n	**Baseline score mean (SD)**	**Change from baseline (all postbaseline) LS mean scores (SE)**	**LS mean change difference (SE) [95% CI] across treatments**
**Index Score[a]**				
Abemaciclib + NSAI	35	0.77 (0.20)	-0.00 (0.02)	-0.02 (0.04) [-0.1, 0.07]
Placebo + NSAI	15	0.78 (0.12)	0.01 (0.04)	
**EQ-5D VAS[b]**				
Abemaciclib + NSAI	36	73.06 (20.71)	-4.29 (2.17)	-0.38 (3.96) [-8.31, 7.57]
Placebo + NSAI	15	77.60 (12.04)	-3.92 (3.30)	

United Kingdom value set used
[a]Overall index score where 1 represents best possible health and 0, death
[b]EQ-5D VAS represents a single self-rated score ranging from 100 (best imaginable health state) to 0 (worst imaginable health state)

CI, confidence interval; *EQ-5D-5L*, EuroQoL-5 dimension-5 level version; *LS*, least squares; *n*, number of patients in the intent-to-treat population with baseline and postbaseline scores; *SD*, standard deviation, *SE*, standard error; VAS, visual analog scale.
